# Endogenous Pituitary Adenylate Cyclase-Activating Polypeptide (PACAP) Plays a Protective Effect Against Noise-Induced Hearing Loss

**DOI:** 10.3389/fncel.2021.658990

**Published:** 2021-03-22

**Authors:** Jérôme Ruel, Matthieu J. Guitton, Paul Gratias, Marc Lenoir, Sanbing Shen, Jean-Luc Puel, Philippe Brabet, Jing Wang

**Affiliations:** ^1^Institute for Neurosciences of Montpellier (INM), University Montpellier, INSERM, Montpellier, France; ^2^Laboratoire de Neurosciences Cognitives, UMR7291 CNRS, Aix-Marseille Université, Marseille, France; ^3^CERVO Brain Research Center, Faculty of Medicine, Laval University, Quebec City, QC, Canada; ^4^Regenerative Medicine Institute, National University of Ireland (NUI), Galway, Ireland

**Keywords:** acoustic trauma, PACAP (pituitary adenylate cyclase-activating polypeptide), PAC1 receptor, neuroprotection, noise-induced hearing loss (NIHL)

## Abstract

Pituitary adenylyl cyclase-activating polypeptide (PACAP) is a member of the vasoactive intestinal polypeptide (VIP)-the secretin-glucagon family of neuropeptides. They act through two classes of receptors: PACAP type 1 (PAC1) and type 2 (VPAC1 and VPAC2). Among their pleiotropic effects throughout the body, PACAP functions as neuromodulators and neuroprotectors, rescuing neurons from apoptosis, mostly through the PAC1 receptor. To explore the potential protective effect of endogenous PACAP against Noise-induced hearing loss (NIHL), we used a knockout mouse model lacking PAC1 receptor expression (PACR1^−/−^) and a transgenic humanized mouse model expressing the human PAC1 receptor (TgHPAC1R). Based on complementary approaches combining electrophysiological, histochemical, and molecular biological evaluations, we show PAC1R expression in spiral ganglion neurons and in cochlear apical cells of the organ of Corti. Wild-type (WT), PAC1R^−/−^, and TgHPAC1R mice exhibit similar auditory thresholds. For most of the frequencies tested after acute noise damage, however, PAC1R^−/−^ mice showed a larger elevation of the auditory threshold than did their WT counterparts. By contrast, in a transgene copy number-dependent fashion, TgHPAC1R mice showed smaller noise-induced elevations of auditory thresholds compared to their WT counterparts. Together, these findings suggest that PACAP could be a candidate for endogenous protection against noise-induced hearing loss.

## Introduction

Noise-induced hearing loss (NIHL) is a common cause of hearing impairment in industrialized countries (Robinson et al., 2015; Varela-Nieto et al., 2020). Brief and mild acoustic overexposure can lead to temporary hearing loss characterized as a temporary threshold shift (TTS) of auditory responses, while exposure to high-intensity sound or repeated overstimulation can trigger irreversible increases in hearing thresholds, leading to a permanent threshold shift (PTS; Wang et al., 2007; Fetoni et al., 2013; Wang and Puel, 2018). To date, the exact mechanisms driving noise-induced hearing loss remain only partially understood. This is probably due to the complexity of intrinsic (genetic predisposition, aging) and extrinsic (e.g., leisure or occupational setting) factors, and of their interactions (Śliwińska-Kowalska and Zaborowski, 2017).

Pituitary adenylyl cyclase-activating polypeptide (PACAP), is a member of the vasoactive intestinal polypeptide (VIP)—the secretin-glucagon family of neuropeptides with anti-inflammatory properties (Martínez et al., 2006; Vaudry et al., 2009; Hirabayashi et al., 2018). PACAP appears in two biologically active forms, PACAP38 containing 38 amino acids, and PACAP27 consisting of the N-terminal 27 amino acid residues of PACAP38 (Miyata et al., 1990). They act through two classes of receptors: PACAP type 1 (PAC1) and type 2 (VPAC1 and VPAC2; Vaudry et al., 2000b, 2009; Hannibal et al., 2017; Hirabayashi et al., 2018). Among their pleiotropic effects throughout the body, PACAP functions as neuromodulators and neuroprotectors, notably contributing to the rescue of neurons from apoptosis (Vaudry et al., 2000a; Dohi et al., 2002; Fukushima et al., 2002; Hamelink et al., 2002; Dejda et al., 2005; Stumm et al., 2007; Cheng et al., 2018) and decreasing glutamate-induced neurotoxicity (Onoue et al., 2002; Stumm et al., 2007; Kaneko et al., 2018) through the PAC1 receptor.

To explore the potential protective effect of endogenous PACAP against noise-induced hearing loss, we used a knockout mouse model lacking PAC1R expression in a C57BL/6J background (C57BL/6J PAC1R^−/−^; Jamen et al., 2000), and a transgenic humanized mouse model expressing human PAC1R (TgHPAC1R) in a CBA × C57BL/6J background (Lang et al., 2006). Based on complementary approaches combining electrophysiological, histochemical, and molecular biological evaluations, we show that Adcyap1r1, the gene encoding PAC1R, is intrinsically expressed in auditory neurons and that its deletion contributes to vulnerability to acoustic trauma. The expression of human PAC1R, however, rescues hearing, thus suggesting that endogenous PACAP plays a protective effect against noise-induced hearing loss.

## Materials and Methods

### Animals

Mice deficient in PAC1 receptor (PAC1R^−/−^) were obtained by gene targeting as previously described (Jamen et al., 2000). Congenic PAC1R^+/–^ strains were generated from back-crossing for ten generations with C57BL/6 inbred strains. PAC1R^+/–^ mice were crossed to produce F2 PAC1R^−/−^ mice and Wild-type (WT) littermates, which served as controls. Transgenic mice expressing human PAC1R (TgHPAC1R) were generated and maintained on a mixed genetic background of CBA × C57BL/6J as described before (Shen et al., 2000). The TgHPAC1R mice used in the present study were produced by crossing heterozygous offspring of the Tg founder with two to six copies of the transgene (Lang et al., 2006). Animals were housed in pathogen-free animal-care facilities accredited by the French Ministry of Agriculture and Forestry (C-34-172-36; December 19, 2014). Experiments were carried out following French Ethical Committee stipulations regarding the care and use of animals for experimental procedures (agreements C75-05-18 and 01476.02, license #6711). All experimental procedures were conducted with 2–3 month-old male mice.

### Genotyping

The Primers used for genotyping of PAC1R^−/−^ were previously described (Jamen et al., 2000): forward primer exon 7 (5′-TGGGTTTGATGACTATGAGC-3′), reverse primer exon 8 (5′-TGAGGGTGACGAGGGAGGTG-3′), and neomycin resistance reverse primer (5′-GCCTTCTATCGCCTTCTTGA-3′). The PCR products are 591 bp (WT) and 394 bp (mutant). Genotyping of TgHPAC1R mice was performed with two pairs of primers. The human *ADCYAP1R1*-specific primers were derived from the intron region after the Hop exon. The TgHPAC1For forward primer is 5′-GTTGGAGATTGCCGATGCC-3′ and the reverse TgHPAC1Rev primer is 5′-TCAGTCAATAGCCTGTAGAACC-3′; the product is 389 bp. A pair of primers from the Intron two region of the mouse *ADCYAP1R1* gene was used to amplify a 605 bp PCR product as an internal control. The mPAC1In2For primer is 5′-ACTCTCAGTGACATTAGGTGGC-3′ and the mPAC1In2Rev primer is 5′-CAGGCAGATGGTTATTGAGTCC-3′.

### Expression of the PAC1R Splice Variant mRNA in the Cochlea

Cochleae were mechanically dissected from 2-month-old WT C57BL/6 mice and immediately processed for total RNA isolation using the RNeasy Mini protocol. One microgram of total RNA was reverse transcribed and PCR amplification was then performed with specific primers as described (Jamen et al., 2002). To identify all PAC1R splice variants, we used primers flanking the N-terminal extracellular domain and the hip-hop intracellular loop. Total RNA from the mouse cerebellum was processed in parallel for sample comparison. The PAC1R PCR products were purified and sequenced with the same primers. PCR analysis required two animals per sample and was performed in biological and technical triplicate.

### Cellular Localization of the HPAC1R Transgene in the Cochlea

Adult TgHPAC1R and WT mice (*n* = 3 per strain) were killed with a lethal dose of sodium pentobarbitone and perfused transcardially with a cold 4% paraformaldehyde solution and then with PBS to wash out the fixative. Both cochleae were removed and the bony capsules of each cochlea were dissected and stained overnight at 37°C with X-Gal buffer [1 mg/ml 5-Bromo-4-chloro-3-indolyl-D-galactoside, 5 mM potassium ferricyanide, 5 mM potassium ferrocyanide, 2 mM MgCl_2_, 0.02% NP-40 in phosphate-buffered saline (PBS)]. Cochleae were then washed with PBS. Reissner membrane was removed to expose the organ of Corti and observed under a binocular loupe (Leica m80). For the flat preparations, the cochlea was divided into two parts from apex to base. The surface of each cochlear turn was exposed by removing the tectorial membrane. Whole mounts of cochlear segments were observed on a microscope (Upright 1, Leica DMRB).

### Acoustic Trauma

Animals were placed under anesthesia using an intraperitoneal injection of a mixture of Rompun 2% (3 mg/kg) and Zoletil 50 (40 mg/kg) and exposed to a continuous 6 kHz pure tone of 130 dB SPL for 20 min. Tones were generated by a waveform synthesizer (Hewlett-Packard 8904A, Palo Alto, CA, USA), and presented to the ears in the free field *via* a JBL 075 earphone (JBL, Northridge, CA, USA) positioned 10 cm in front of the animal’s head. The sound level was measured using a calibrated 1/2” Brüel and Kjaer microphone (Brüel and Kjaer 4314, Atlanta, GA, USA) and a Brüel and Kjaer calibrating amplifier (Brüel and Kjaer 2606, Atlanta, GA, USA).

### Recording of Compound Action Potentials (CAPs) of the Auditory Nerve

Functional evaluations were performed in animals under anesthesia before and 20 min after noise exposure (*n* = 8–14 per strain). Animals were placed on an anti-vibration table (TMC, Peabody, MA, USA) inside a Faraday-shielded, anechoic, sound-proof cage. The rectal temperature was measured with a thermistor probe and maintained at 38°C ± 1°C using a heated blanket. Using a retro-auricular surgical approach, a silver-wire electrode was placed on the bony edge of the round window niche, and an intradermal needle in the mastoid muscles. The acoustical stimuli were delivered under calibrated conditions using a custom acoustic assembly comprising a signal generator (PXI-4461 controlled by LabVIEW, National Instrument Company), an audio amplifier (Tucker Davis, SA1), and a magnetic speaker (Tucker Davis, MF1). Cochlear compound action potentials (CAP) to 10 ms-tone bursts (1 ms rise and fall) of frequencies from 2 to 32 kHz and sound pressure levels from 0 to 100 dB SPL were amplified (2,500 times, VIP-20 amplifier), sampled (at a rate of 50 kHz), filtered (bandwidth of 0.3–3 kHz), and averaged (100–700 times). Data were displayed using LabView software and stored on a computer (Dell T7400). Auditory thresholds were defined as the lowest sound intensity that elicited a clearly distinguishable response.

### Statistical Analysis

Data are expressed as the mean ± SEM. The normality of the variables was assessed using the Shapiro-Wilks test. The significance of the group difference was assessed with an unpaired Student’s *t*-test using SigmaPlot 13.0 software.

## Results

### Endogenous PAC1R Gene Expression in the Mouse Cochlea

**Figure 1:**
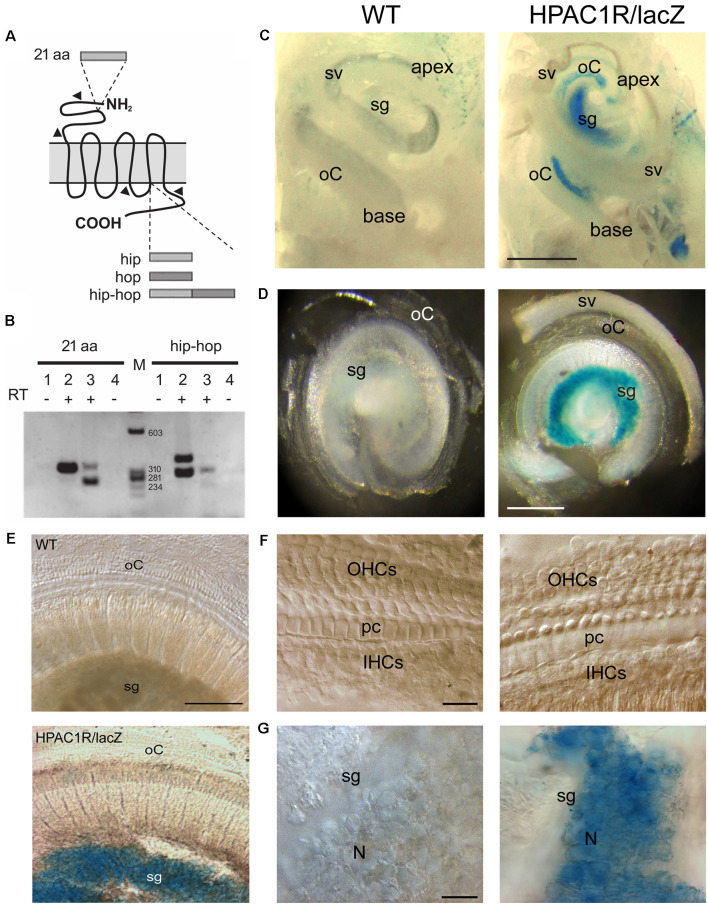
Endogenous PAC1R gene expression and human PAC1R transgene localization in the mouse cochlea. **(A)** Schematic of PACAP TYPE 1 (PAC1) receptor splicing variants. Two splicing sites have been demonstrated. A 21 aa cassette in the extracellular N-terminal binding domain of pituitary adenylate cyclase-activating polypeptide (PACAP) and hip and hop cassettes in the third intracellular G-protein interacting domain. **(B)** Total RNA was reverse-transcribed and PCR amplified with primer pairs specific for PAC1 isoforms (arrowheads in **A**). Lanes 1 and 4: CTL PCR without RT; Lanes 1, 2: Cerebellum; lanes 3, 4: Cochlea, M: size markers. PCR analysis required two animals per sample and was performed in biological and technical triplicate. **(C–G)** Representative micrographs of X-gal staining showing dissected mouse cochleae **(C)**, flat preparations of the basal part of the cochleae **(D,E)**, and higher magnification of organs of Corti **(F)** and spiral ganglia **(G)** of wild type (WT; left column in **C,D,F**, and **G**) and HPAC1R/lacZ Tg (right column in **C,D,F**, and **G**) mice (*n* = 3 per strain). HPAC1R expression is visualized using a *LacZ* marker (blue). Note that transgene expression is prominent in the spiral ganglion (sg) region throughout the cochlea. oC, organ of Corti; sv, *stria vascularis*; OHCs, outer hair cells; pc, pillar cells; IHCs, inner hair cells; N, neuron. Scale bars: **(C)** = 500 μm, **(D)** = 300 μm, **(E)** = 120 μm, **(F)** = 15 μm, **(G)** = 30 μm.

Total RNA isolated from 2-month-old mouse cochleae were analyzed by RT-PCR for detection of PAC1R splice variants with extracellular (21 aa) and intracellular (“hip and “hop”) alternative domains (Figure 1A). Two variants of PAC1R, which differ in the N-terminal domain (Figure 1B, left lane 3, 269 bp and 332 bp bands), but without an intracellular cassette (Figure 1B; right lane 3, 305 bp band), were found to be specifically expressed in the cochlea. Interestingly, the shortest mRNA form (269 bp band) encoding a receptor without the 21 aa appeared predominant in the cochlea. Mouse cerebellum, here used as a positive control (Figure 1B, lane 2, 332 bp band), is known to express PAC1R variants with the 21 aa domain (Jamen et al., 2002).

### Human PAC1R Transgene Localization in the Mouse Cochlea

TgHPAC1R mice expressed the human PAC1R in a manner closely resembling the endogenous expression pattern (Lang et al., 2006). Helpfully, the detection of the transgene expression was facilitated by an IRES-lacZ reporter gene incorporated at the 3’UTR of the human *Adcyap1r1* gene. In whole-mount staining of the cochleae, intense, specific lacZ positive cells with blue cytosol were observed mostly in the spiral ganglion region throughout the cochlea, from the basal to the apical turn of TgHPAC1R mice (Figures 1C–G), compared with WT mice (Figures 1C–G). Additional weaker X-gal stained lacZ positive cells were also observed within the organ of Corti of the apical part of the cochlea (Figure 1C). Observations at a higher magnitude of the basal turn (Figures 1F,G) clearly show restricted X-gal staining in the spiral ganglion neurons. These results established a very high level of expression of PAC1R in spiral ganglion neurons and a lesser expression in the organ of Corti of the apical turn. Moreover, no specific lacZ positive cells were observed in the stria vascularis (Figures 1C,D).

### PAC1R Deletion Exacerbates Noise-Induced Hearing Loss, While Its Overexpression Protects the Cochlea

**Figure 2:**
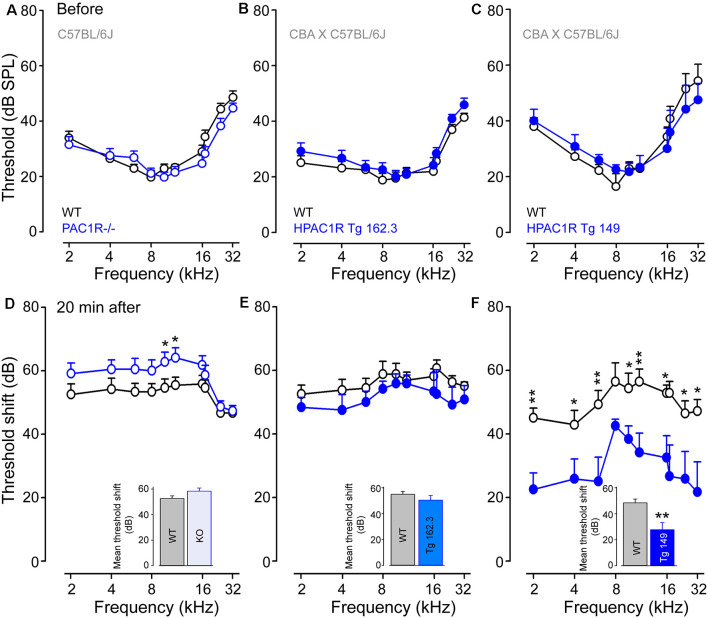
PAC1R deletion exacerbates noise-induced hearing loss (NIHL), while its overexpression protects the cochlea. **(A–C)** Compound action potentials (CAPs) thresholds recorded before noise exposure in WT littermates (black plot, **A**–**C**), PAC1R^−/−^ (empty blue plot, **A**), TgHPAC1R 162.3 (filled blue plot, **B**) and TgHPAC1R 149 (filled blue plot, **C**). **(D–F)** Noise-induced threshold shift in WT (black plot in **D–F**), PAC1R^−/−^ (empty blue plot in **D**), TgHPAC1R 162.3 (filled blue plot in **E**), and TgHPAC1R 149 (filled blue plot in **F**) mice 20 min after noise exposure. Note that hearing thresholds before noise exposure were similar in genetically modified mice and their WT littermates. At 20 min after exposure, an enhanced noise effect was seen in PAC1R^−/−^ mice, whereas a significant protective effect was observed in TgHPAC1R 149 mice compared with WT mice. Data are means ± SEM (*n* = 8–14 per strain). Unpaired Student’s *t*-test, *P ≤ 0.05, ** *P* ≤ 0.001. **Inset** in (**D–F**): Mean threshold shifts from 2 kHz to 32 kHz in PAC1R^−/−^ **(D)**, TgHPAC1R 162.3 **(E)**, and TgHPAC1R 149 **(F)**, and their WT mice 20 min after noise exposure.

Auditory thresholds before, and 20 min after, noise exposure were measured by recording the tone-burst-evoked compound action potential (CAP), which captures the synchronous activation of the auditory nerve fibers, and is commonly used to probe deafness in experimental and clinical settings. Our results showed that CAP thresholds of PAC1R^−/−^, TgHPAC1R 162.3 (carrying two copies of the human transgene) and TgHPAC1R 149 (with six copies of the transgene) were virtually identical to their WT littermates before noise exposure (Figures 2A–C).

The intense sound exposure induced relatively flat threshold losses of about 55 dB between 1 and 20 kHz for all WT strains. PAC1 receptor deletion slightly, but significantly (*p* < 0.05 at 10 and 12 kHz), exacerbated noise-induced threshold shifts compared with the WT (mean threshold shifts: 52.6 ± 1.1 dB and 58.3 ± 1.7 dB in WT and PAC1R^−/−^ mice, respectively) (Figure 2D). Interestingly, overexpression of human PAC1R prevented the acute noise-induced hearing loss in a transgene copy number-dependent manner (mean threshold shifts for Tg 162.3: 56.5 ± 0.8 and 51.7 ± 1.0 dB in WT and Tg mice, respectively; mean threshold shifts for Tg 149: 50.4 ± 1.5 and 29.1 ± 2.2 dB in WT and Tg and mice, respectively) (Figures 2E,F). Maximum protective effects were observed in Tg 149 mice, which carry six copies of the human PAC1R transgene (Figure 2F).

## Discussion

This study reports for the first time a protective role of endogenous PACAP against noise-induced hearing loss in mammals. We show that PAC1R is mainly expressed in spiral ganglion neurons, and, to a lesser extent, in the organ of Corti of the apical turn of WT C57BL/6 mice. PAC1R deletion in this strain mice enhanced noise-induced hearing loss. By contrast, overexpression of human PAC1R in CBA × C57BL/6J mice reduced the acute effects of acoustical trauma in a transgene copy number-dependent manner.

### Expression and Localization of PACAP and PAC1R in the Cochlea

PACAP expression in the rat cochlea was initially demonstrated at the mRNA level using *in situ* hybridization (Kawano et al., 2001). PACAP mRNA was observed in the cytoplasm of most cells in the spiral ganglion and in marginal cells in the stria vascularis. Later, analyses of rat cochlear subfractions by RT-PCR revealed multiple splice variants of PAC1-R in different locations of the cochlea. There are five PAC1-R splice variants in the lateral wall, two in the organ of Corti, and one in the spiral ganglion (Abu-Hamdan et al., 2006). Consistent with these previous studies, we demonstrate here that two variants of PAC1R, which differ in the N-terminal domain but without an intracellular cassette, are specifically expressed in the mouse cochlea.

Within the lateral wall, the neuropeptide was immunolocalized primarily to the stria vascularis in the basolateral extensions of marginal cells. Within the organ of Corti and the spiral ganglion, PACAP was also found in efferent nerve fibers, as well as in axons of afferents leaving the spiral ganglion and joining the auditory nerve (Drescher et al., 2006). Also, Tamas et al. (2012) found that both inner and outer hair cells, as well as the outer phalangeal cells (Deiters’ cells), showed PAC1-R expression and suggested that PACAP may play a role in calcium homeostasis. Our results on X-gal staining in Tg mice indicate a very high level of expression of the human PAC1R transgene in auditory neurons and a lower expression in the organ of Corti of the apical turn, but not in the stria vascularis. One possible explanation for the difference in the expression profiles of PAC1R observed in our study and others (Abu-Hamdan et al., 2006) might have resulted from the differences in the gene structure between the human and mouse loci leading to PAC1R expression in stria vascularis undetectable in TgHPAC1R mice but present in the rat cochlea. Another explanation could be that this expression in the stria vascularis was transient in young animals but absent in the adult stage. Since Abu-Hamdan et al. (2006) used 20-day old ACI Black Agouti rats and we used 2–3 months-old mice. A third explanation would be that the lateral wall is described as the main source of transcription of the PAC1R splice variant in the cochlea, whereas our RT-PCR analysis in the whole cochlea allows us to detect only the PAC1R-short and PAC1R-null variants, both without the intracellular variant. We can assume that their level of expression remains relatively low in the stria vascularis compared to the other cochlear structures.

### Protective Effect of PACAP and PAC1R in the Cochlea

Using noise-exposed mice with PACAP deletion or over-expression of the human PACAP transgene, we identified a protective effect of endogenous PACAP against acute noise-induced hearing loss. Interestingly, a recent study demonstrated that endogenous PACAP is essential in the maintenance of hearing during aging in mice (Fulop et al., 2019). Also, previous results from the same lab have already demonstrated an anti-apoptotic effect of PACAP against oxidative stress-induced chicken cochlear cell death *in vitro* (Racz et al., 2010). Furthermore, they demonstrated that the levels of Ca^2+^-binding proteins (parvalbumin, calretinin, calbindin) are elevated in the hair cells of PACAP KO animals compared to WT ones under normal circumstances (Nemeth et al., 2014). Treatment with the ototoxic aminoglycoside kanamycin induced elevated Ca^2+^-binding protein levels in WT animals, but not in PACAP KO animals.

Together, our results and others highlight the need to probe the possibility of using exogenous PACAP or an agonist of its receptors to treat age-related or noise-/ototoxic-drug-induced hearing loss. However, particular caution should be taken regarding using exogenous PACAP polypeptide and receptors for any therapeutic application. Since PACAP and its receptors may mediate opposing effects in the different pathophysiological conditions such as migraine, post-traumatic stress disorder, ischemia, Alzheimer’s disease, cancers, and inflammation (Denes et al., 2019).

### Potential Mechanisms Underlying the Neuroprotective Effect of PACAP in the Cochlea

We have already shown that the noise paradigm (6 kHz, 130 dB SPL, 15 min) that we used in the present study induced massive apoptotic death of the cochlear hair cells and the swelling of auditory nerve terminals at their hair-cell synapses as early as 6 h after noise exposure (Wang et al., 2007). The hair cell apoptosis may maintain for days leading to a permanent hearing loss in guinea pigs (Wang et al., 2007). Several lines of evidence revealed also noise trauma contributed to the immediate presence of apoptosis in the murine central auditory pathway and later occurrence of central auditory structural disorders such as neuronal reorganization, hyperactivity, and hyperexcitability (Komiya and Eggermont, 2000; Gröschel et al., 2014, 2018). These central deficiencies may be one of the major causes of chronic tinnitus, hyperacusis, impaired speech processing, and central neurodegeneration (Komiya and Eggermont, 2000; Gröschel et al., 2014, 2018).

Here, we showed that the endogenous PACAP plays a protective effect against noise-induced acute hearing loss. Even though the actual extent of the mechanisms underlying the neuroprotective effect of PACAP observed in the present study is still not known. Given the complex and numerous roles of PACAP on both neurotransmission and cellular functioning in various systems, it is likely that the neuroprotective effect of PACAP against noise-induced hearing loss is multifactorial.

First, the fact that human PAC1R transgene mainly localized in auditory neurons suggests that some of the protective effects of PACAP observed in TgHPAC1R mice might be mediated by these receptors at the sensory neuron level, acting as a stress response peptide that is necessary for protection against different insults, as demonstrated in ethanol- or oxidative-insulted cerebellar neurons (Vaudry et al., 2005) and NMDA-induced retinal damage (Endo et al., 2011). PACAP may also act on the injured neuron by binding to PAC1 receptors and subsequently activating cAMP-dependent protein kinase A and mitogen-activated protein (MAP) kinase pathways (Vaudry et al., 1998; Shoge et al., 1999; Silveira et al., 2002). In this context, *in vitro* studies have demonstrated that PACAP treatment protects neurons against various cytotoxic agents, including glutamate-induced cytotoxicity (Vaudry et al., 1998; Shoge et al., 1999; Silveira et al., 2002).

Second, immunoreactivity for both PACAP and PAC1-R was associated within the marginal cells of the stria vascularis (Abu-Hamdan et al., 2006), PACAP may exert its protective effect in an autocrine/intracrine fashion, as previously demonstrated in cultured rat cortical neurons exposed to glutamate (Shintani et al., 2005).

Third, PACAP could also act more distally and indirectly. Indeed, it has been observed that PACAP, in addition to direct stimulation of neurons, can also affect them indirectly *in vivo*, notably through the stimulation of release of neuroprotective factors including neurotrophin-3, activity-dependent neurotrophic factor, by surrounding astrocytes (Masmoudi-Kouki et al., 2007; Stumm et al., 2007; Kong et al., 2016).

We observed that X-gal staining in TgHPAC1R mice was mainly restricted to the primary auditory neurons of the mouse cochlea and absent from hair cells. However, a previous study has already demonstrated that PACAP is localized in efferent nerve fibers, as well as in axons of afferents leaving the spiral ganglion and joining the auditory nerve (Drescher et al., 2006). We thus speculate that PACAP might also act on afferent dendrites after its release from lateral efferent nerve endings. Also, an elevation of Ca^2+^-binding proteins was observed in PACAP KO animals and kanamycin exposed WT mouse cochleae, therefore, endogenous PACAP might be a compensatory mechanism indicating pathological conditions in the inner ear (Nemeth et al., 2014).

Currently, it cannot be ruled out that PACAP would be able to act on other structures controlling cochlear liquid homeostasis through other molecular pathways than those involving PAC1R (Lerner-Natoli et al., 1997). Interestingly, in the whole cochlea of adult mice, we detected two major PAC1R mRNA splice variants: PAC1-null and PAC1-short variants. The PAC1-short variant is the predominant variant that we found in the mouse cochlea. The splicing of these exons 5 and 6 encoding the 21aa extracellular domain was related to two important pharmacological features (Blechman and Levkowitz, 2013): one is that the receptor affinity to PACAP-27 and VIP is significantly higher and unchanged for PACAP-38 (Pantaloni et al., 1996; Dautzenberg et al., 1999). Second, these changes in binding affinity account for an increase in the PACAP-27 and VIP potencies to stimulate inositol phosphate production (Pantaloni et al., 1996; Lutz et al., 2006). Therefore, it could be of interest to test whether VIP and PACAP have similar potencies to protect auditory neurons against noise- ototoxic drug-induced and age-related hearing loss.

## Conclusion

The large intersubject variability in acoustic injury is well known. The exact mechanisms underlying these differences remain, however, poorly understood. Therefore, identifying genes of vulnerability or genes that could potentially play a protective role is critical to enhancing the development of personalized medicine for hearing loss. In this context, PACAP may be a candidate gene for endogenous protection against noise-induced hearing.

## Data Availability Statement

The original contributions presented in the study are included in the article, further inquiries can be directed to the corresponding author.

## Ethics Statement

The animal study was reviewed and approved by French Ethical Committee stipulations regarding the care and use of animals for experimental procedures (agreements C75-05-18 and 01476.02, license #6711).

## Author Contributions

JW, MG and PB designed the experiments. SS provided transgenic mice expressing human PAC1R. JW, MG and JR performed electrophysiological assessments. JW and ML performed histochemical evaluations. PB performed molecular biological assessments. PG carried out quantitative analysis. JW, MG and PB wrote the manuscript. J-LP and JR reviewed the manuscript. All authors contributed to the article and approved the submitted version.

## Conflict of Interest

The authors declare that the research was conducted in the absence of any commercial or financial relationships that could be construed as a potential conflict of interest.
